# Medication impact on oral health in schizophrenia

**DOI:** 10.4317/medoral.26061

**Published:** 2023-11-22

**Authors:** Leire Urien, Nerea Jauregizar, Unax Lertxundi, Unai Fernández, Teresa Morera-Herreras

**Affiliations:** 1 Department of Pharmacology, Faculty of Medicine and Nursing, University of the Basque Country (UPV/EHU), Leioa, Spain; 2 Bioaraba Health Research Institute; Osakidetza Basque Health Service, Araba Mental Health Network, Araba Psychiatric Hospital, Pharmacy Service, Vitoria-Gasteiz, Spain; 3 Neurodegenerative diseases Group, Biocruces Health Research Institute, Barakaldo, Bizkaia, Spain

## Abstract

**Background:**

Patients with schizophrenia constitute a particularly vulnerable group for oral diseases. Among the different factors involved, we aimed to examine the evidence of how drugs could contribute to the poorer oral health of this population.

**Material and Methods:**

An overview of the potential impact of medication on dental/oral health among people with schizophrenia was proposed focusing on selected literature.

**Results:**

Studies show a higher dental caries and degree of periodontal diseases in this population and point to drug-induced xerostomia as an important risk factor for oral health deterioration. The risk of dry mouth depends on not only antipsychotics, but also drugs with anticholinergic activity. We hypothesize that antipsychotic induced glycaemic alterations might contribute to reduced oral health, and that the antimicrobial activity of certain antipsychotics could have an impact on oral microbiota affecting oral condition. Pharmacovigilance data show that involuntary movements are caused by typical and some atypical antipsychotics. Dry mouth is most frequently reported for quetiapine and olanzapine, while clozapine is more frequently associated with sialorrhea.

**Conclusions:**

Literature clearly shows higher caries and periodontal disease in schizophrenic patients. However, overall, there is scarce literature about the potential influence of drugs in these disorders. Health professionals should be aware of this issue in order to implement adequate preventive measures in this vulnerable population.

** Key words:**Schizophrenia, oral health, dental disease, antipsychotics, anticholinergic burden.

## Introduction

Among psychiatric disorders, schizophrenia is probably the illness with the greatest impact on people's quality of life and associated disability. Patients with schizophrenia have higher average mortality rates and physical comorbidity than the general population. It has been widely shared that mental disorder-related factors, inequalities in access to health care, unhealthy lifestyles and also psychotropic medications can contribute to the onset or exacerbation of physical illnesses (1). One of the least studied physical health areas among these patients is oral health (2-4), even if the few studies available on this subject suggest that these patients have overall poorer oral health status in terms of dental caries and periodontal disease (2-6). Moreover, oral disease can have important consequences in patients with schizophrenia, like social withdrawal, low self-esteem and chronic medical diseases such as cardiovascular conditions (7).

Different potential risk factors have been related to the poorer oral health of this population, including unhealthy lifestyle (poor oral hygiene and inadequate nutrition, consumption of alcohol, abuse substances, tobacco and sugary drinks), systemic diseases (obesity, metabolic syndrome or diabetes mellitus), and difficulties in accessing dental care (8-9). Additionally, antipsychotic medications and other psychotropic drugs can also cause or aggravate dental diseases, including salivary secretion dysfunctions, which in turn, may trigger periodontal disease, among others (3,4,6). However, to date very few studies have investigated the link between antipsychotic medication and poorer oral health of patients with schizophrenia. Some authors suggest that the contribution of psychotropic drugs to xerostomia should be taken into account when prescribing, which requires a greater collaboration between health professionals (6). Additionally, the risk of dry mouth in a certain individual depends on not only antipsychotics, but also many other drugs with anticholinergic activity. In this line, the estimation of the total anticholinergic burden of a certain patient and its potential impact on oral health deserves study.

Aware of the importance of this issue, efforts are being made to improve the public health from a preventive care perspective. For instance, interventions in Australia include the Queensland´s strategy to improve the physical health of people with severe mental illness. Similarly, a dental check-up is recommended in France since 2016 for all new admissions to a psychiatric hospital (3).

Therefore, it is of paramount importance to inform health professionals about the association between the medication and oral cavity diseases that may suffer their patients with schizophrenia. The aim of this review is to update clinicians about the current clinical evidence that supports or suggests the association between dental and oral diseases and antipsychotics.

## Material and Methods

A literature search of the MEDLINE database (via Pubmed) for articles published from 1990 to march 2021 was conducted. To perform the search, the following MeSH terms were used, combining them alternately: “"schizophrenia" OR "mental illness" AND "oral health" OR "poor oral health" OR "dental care" OR "dental caries" OR "tooth loss" OR "periodontitis" OR "advanced dental disease" OR "antipsychotic drugs" OR "anticholinergic drugs". In addition to the articles identified through this search strategy, 7 additional studies identified through the bibliographic references of the articles selected in the first phase of the search were selected. Eligibility criteria were mainly studies examining the oral health of people with a diagnosis of schizophrenia, both inpatient and outpatient, of any age and gender. Exclusion criteria included: 1) other mental disorders as primary psychiatric diagnosis (except for the evaluation of the rate of edentulism); 2) results without DMFT index; 3) studies without a control group; 4) survey studies, case series, systematic reviews and meta-analyses, as well as animal studies.

In addition, in order to investigate the possible relationship between oral health and antipsychotic medication, a study was conducted using data obtained from the European Pharmacovigilance database, EudraVigilance (http://www.adrreports.eu/-European database of suspected adverse drug reaction reports), from which searches for suspected adverse reactions of the oral cavity caused by antipsychotic drugs were performed. For this, the antipsychotic drugs amisulpiride, aripiprazole, asenapine, brexpiprazole, cariprazine, clozapine, lurasidone, olanzapine, paliperidone, quetiapine, risperidone and ziprasidone were selected. We then selected the search option "number of individual cases for a selected reaction" and restricted the search to "gastrointestinal disorders" in the time period from 1 January 2012 to 1 March 2021.

## Results

- Dental and oral health among people with schizophrenia

So far, research about oral health in people with schizophrenia has been focused on two main diseases: dental caries (tooth decay) and periodontal disease (gum disease).

Dental status in schizophrenia

While clinical investigations on dental status in psychiatric patients have increased in recent decades, there is very scant evidence about the dental health specifically in schizophrenic patients compared with the rest of the population (Table 1) (2,5,6,10-14). In all of these few studies, the dental condition was determined according to the standardized criteria of the Decayed, Missing and Filled teeth (DMFT) index and component scores. DMFT is the sum of decayed, missing due to caries and filled teeth in the permanent teeth. Therefore, a high DMFT index score indicates worse dental health. Except in the study by Arnaiz *et al*. (2), people with schizophrenia considered were inpatients. In principle, in a worst psychopathological state than not hospitalized patients and, accordingly, corresponding to worst self-care and personal hygiene in relationship with negative symptomatology (15).

**Table 1:**
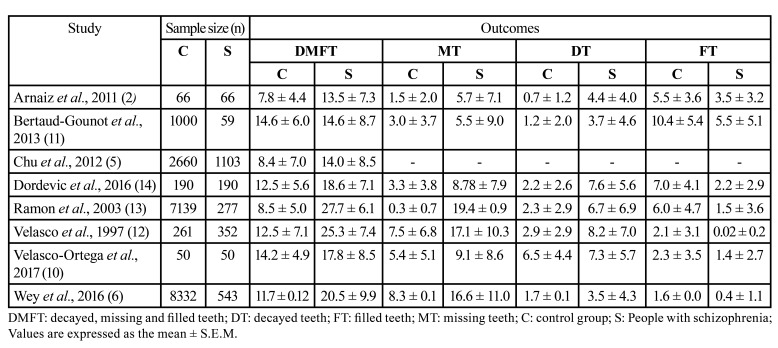
Decayed, missing and filled teeth (DMFT) index and component scores in schizophrenic patients and control group.

Data from these studies reveal that people with schizophrenia show a DMFT index significantly higher compared to the general population (between 13.5 and 27.7 versus 7.8-14.6, depending of the studies). More specifically, the decayed (DT) and missing teeth (MT) scores are higher in schizophrenic patients than in the general population, while the rate of filled teeth (FT) is lower. In addition, some studies provided the rate of edentulous (condition wherein a patient has complete tooth loss or just had teeth indicated for dental extraction) which range from 4-5% (5,11) to 11.2-14.2% in schizophrenic people (6,12,13). These data indicate poorer oral health and reduced asses to dental care and pointing out people with schizophrenia as a vulnerable population group.

- Periodontal disease in schizophrenia

The term periodontal disease refers to the common inflammatory disorders of gingivitis and periodontitis that are caused by pathogenic microflora in the biofilm or dental plaque that forms adjacent to the teeth on a daily basis (16). The persistent bacterial infection results in inflammation that can lead to the loosening of teeth, occasional pain and discomfort, impaired mastication, and eventual tooth loss (17).

Schizophrenic patients' periodontal health has been evaluated by using different indexes, although the most used, according to a WHO-recommendation, it has been the community periodontal index (CPI). The CPI evaluates the severity and degree of periodontal diseases by assessing three features (bleeding, subgingival calculus, and periodontal pocket depth) according to 4 levels of severity: 0 (absence of the disease) to 4 (most severe stage). In two of the three available studies, the authors found higher incidence of periodontal disease in people with schizophrenia than in the general population (2,5), while Velasco-Ortega and colleagues described a higher prevalence of calculus (CPI=2) and deep pockets (CPI=4) in control patients (11) (Table 2).

- Pharmacovigilance (EudraVigilance) data

Several disproportionality analyses of pharmacovigilance data from national and international databases have been utilized to investigate the side-effect profiles of antipsychotics. However, a systematic review of such studies found none about their deleterious effects on dental health (18).

In this study, we explored the European pharmacovigilance database (EudraVigilance) (http://www.adrreports.eu/), to look for adverse events of interest associated with antipsychotics related to dental health (Table 3, Table 4). Involuntary movements, affecting the mouth and facial muscles (e.g., tongue protrusion), are caused mostly by typical antipsychotics and some atypical antipsychotics like risperidone and aripiprazole. Apart from being very distressing, these abnormal movements can make dental treatment difficult, with the provision of well-retained dentures especially troublesome.

Dry mouth was most frequently reported for quetiapine and olanzapine both with strong anticholinergic activity (317 and 229, respectively). Clozapine, even if considered a strong anticholinergic, is an exception, since it is more frequently associated with sialorrhea (1.436 notifications). Interestingly, the vast majority of parotid gland swelling cases (33/40) were reported for this drug. This is in line with published literature (19).

**Table 2:**
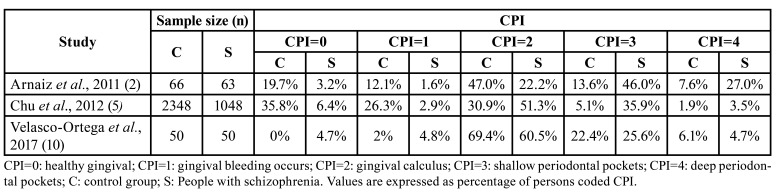
Community periodontal index (CPI) in schizophrenic patients and control group.

**Table 3:**
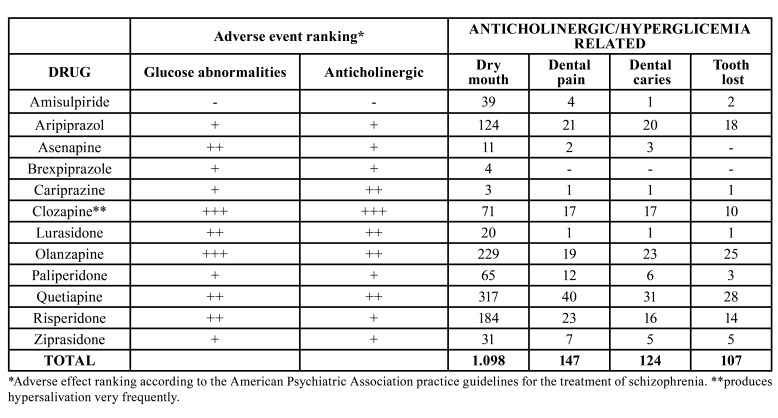
Anticholinergic/hyperglicemia related oral adverse reactions reported with antipsychotic drugs in the European Union pharmacovigilance database (EudraVigilance, 2012-2021).

**Table 4:**
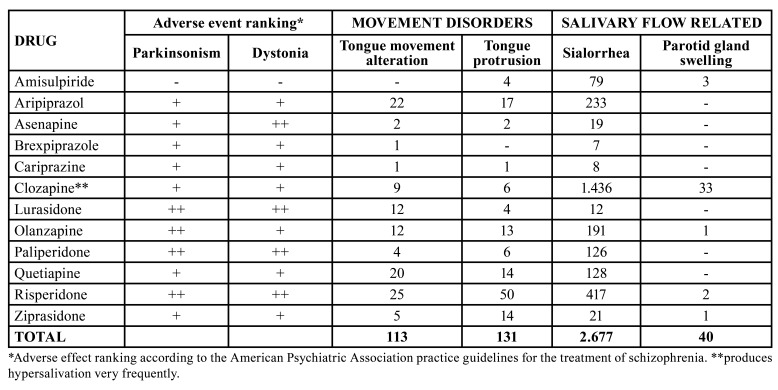
Movement disorders and salivary flow related oral adverse reactions reported with antipsychotic drugs in the European Union pharmacovigilance database (EudraVigilance, 2012-2021).

## Discussion

Poor oral health status in schizophrenic patients has been attributed to multiple factors. Some of them are related to the nature of the disease itself, as the negative symptomatology contributes to demotivation and disinterest in performing appropriate preventive oral hygiene techniques. However, positive symptoms of schizophrenia may also trigger bizarre behaviours leading to self-extractions of teeth and self-injurie in the gingival mucosa (20). Moreover, impaired cognitive functions, such as memory and attentional deficits, reduce the ability of these patients to recognize to own dental treatment needs which could delay the adequate intervention (21). In fact, worse oral health amongst schizophrenic hospitalized patients compared with schizophrenic outpatients with better psychopathological state has been reported, especially in terms of negative symptomatology (10).

On the other hand, low socio-economic status, dental cost, fear and difficulty in accessing the healthcare system or the lack of adequate dental clinics for these patients are the most cited barriers to oral health care (14). In addition, tobacco is a well-known factor for dental health deterioration. It induces increased pocket depth and enhances incidence of tooth loss. The rate of cigarette smoking is high in schizophrenic patients, although decreasing in the last years. However, worldwide prevalence of smoking cessation is still lower in schizophrenic patients than in the general population (22). Additional factors compromising the oral health of these patients could be poor diet (diet high in carbohydrates and low in fibre, sugary drinks), undernutrition and alcohol/substance abuse (8,9).

Nevertheless, we believe that, so far, drugs are an understudied factor that could have a major impact on the oral health in schizophrenia, since they may cause changes in salivary flow and other oral alterations contributing to oral health problems.

- Medication impact on oral health in schizophrenia

The vast majority of people with schizophrenia are under treatment with various psychotropic medications, including antipsychotics, benzodiazepines, antidepressants and others. The side effects of these drugs can include oral disturbances with dental implications in people with schizophrenia.

- Anticholinergic effects of antipsychotics and others drugs used to manage schizophrenia

Several studies have shown that antipsychotic induced xerostomia can contribute to poor oral health in patients with schizophrenia. However, the risk of dry mouth in a certain individual will depend not only on antipsychotics, but also on many other drugs with anticholinergic activity.

- Anticholinergic burden

Anticholinergic risk scales are tools used to estimate the total anticholinergic burden of a certain patient. Such scales usually classify drugs in a range of 0 to 3 points according to their anticholinergic potential. In all scales, the total anticholinergic burden is determined by the sum of the score of each anticholinergic drug. Anticholinergic burden in schizophrenia has been reported as “substantial, common and conferred by multiple medication classes” (23). These patients very frequently suffer from several other comorbidities, which increase the chance of being exposed to additional medications with anticholinergic properties. In the recent study of Joshi *et al*, antipsychotics contributed more than half of the anticholinergic burden, while anticholinergics, antidepressants, mood stabilizers, and benzodiazepines accounted for the remainder (23).

Moreover, patients with chronic psychotic disorders in particular are more vulnerable to prescribing cascades, which in turn may increase the risk and severity of anticholinergic burden. However, so far, studies that have measured anticholinergic burden in schizophrenia have mainly focused on its effects on cognitive decline (24,25). Apart from the effects on the central nervous system, anticholinergic burden has also been associated with heart rate variability reduction in patients with depression and schizophrenia (26), and has also recently been cited in the safety alert issued by the Food and Drug Administration devoted to clozapine induced constipation.

Concerning the possible impact of anticholinergic burden in oral health in this patient population, available information is scarce. An interesting pilot study carried out in France highlighted the potential role of the anticholinergic burden on the oral health-related quality of life (OHrQol). However, the lack of a validated specific scale for the OHrQoL and the lack of concordance among scales to measure anticholinergic burden need to be considered when interpreting their results (27). They used the Anticholinergic impregnation scale (AIS), a scale that considers available drugs in France and is aimed at peripheral anticholinergic activity. These researches showed that patients with higher anticholinergic burden showed lower OHrQoL.

- Lack of concordance of scales to measure anticholinergic burden

A critical problem when interpreting studies measuring anticholinergic burden is the absence of a universally accepted tool to measure it. More than 10 different scales are available, and they differ substantially between them, in both included drugs and the potency of anticholinergic effects assigned to each drug (28). Several studies have shown that different scales or tools used to measure anticholinergic burden in different patient populations yield different results. Concordance between different scales has been generally poor (29,30). To date, as far as we are concerned, no such a study has been conducted specifically in schizophrenic patients, although the first study to report poor concordance between different scales was performed in a Spanish psychiatric hospital (29).

Available online tools like the “Anticholinergic burden calculator” may be helpful for researchers and prescribers (31). Another aspect to keep in mind is that applying drug lists in scenarios that differ from the ones in which they were originally developed can be problematic, as drug availability widely differs among countries. Moreover, to avoid overlooking newer drugs, scales need to be periodically updated. In summary, there is need for good quality validation studies comparing multiple scales to define the best scale.

- Antipsychotics, hyperglycaemia and oral health

Prolonged hyperglycaemia (a characteristic of diabetes mellitus) apart from generating systemic changes, can alter the function of the salivary glands and cause changes in the composition and volume of secreted saliva. Studies have shown decrease in both basal and postprandial salivary flow levels in patients with diabetes (32). This salivary gland hypofunction may lead to alterations at oral mucosa like saburral tongue, periodontal disease, caries, delayed healing of wounds (33).

Apart from their strong anticholinergic activity, some antipsychotics, especially clozapine and olanzapine, have been strongly linked to impaired glucose tolerance, diabetes and diabetic ketoacidosis. Therefore, we hypothesize that antipsychotic induced glycaemic alterations might contribute to dental health deterioration, and that antipsychotics more prone to produce this metabolic alteration may confer a greater risk for dental health deterioration. As far as we are concerned, this particular issue has not been studied and should be a matter of future research.

- Antipsychotics as antibiotics, oral microbiome

Saliva is one of the most important factors that influence the composition of the oral microbial flora. Apart from inducing hyposalivation, antipsychotics might affect dental health in a more indirect, subtle way: i.e., by directly affecting microbial populations. A study published in Nature in 2018 tested >1.000 marketed drugs (from all therapeutic classes) against 40 representative gut bacterial strains. Among the drugs that inhibited the growth of at least one strain, antipsychotics were overrepresented (34). In fact, some phenothiazine drugs have even been suggested for the treatment of antibiotic resistant bacterial strains (35-37). Nevertheless, whether this antimicrobial activity affects oral microbial populations and subsequent apparition of caries remains to be elucidated. It seems that some of the antimicrobial activity occurs at concentrations in the mg/l range, a concentration that may be achieved with the use of orally disintegrating Tablets of certain antipsychotics.

In a study carried in Sweden, the oral microbiota of four groups of patients with hyposalivation of different origin was compared. They found that those patients taking antipsychotics for more than 10 years had an altered number of aciduric and acidogenic microorganisms, similar to patients with Sjögren syndrome (38). Patients on these drugs were also the ones that had the lowest buffer capacity and the only group displaying supragingival plaque. Sadly, no detail about individual antipsychotic use was reported, and the number of patients on antipsychotics (*n*=10) was small.

## Conclusions

Overall, this work shows a worse oral health of people with schizophrenia compared to the general population (with higher prevalence of caries, higher need for tooth extraction...), which raises the importance of ensuring that these people have adequate information and support to live independently, including information on adequate dental care and adequate access to dental care services. Oral health care contributes to improved general health, self-esteem, daily functioning, social inclusion and quality of life, while, conversely, poor oral health predisposes to other physical problems such as respiratory infections or heart disease. Oral health disparity in patients with schizophrenia, including xerostomia, periodontal disease and dental caries, should attract more attention. Accordingly, intervention and prevention programmes should be made available. To get to this point, further studies should be conducted regarding the risk factors that affect the oral health of this population.

Among the factors that can affect the deterioration of oral health in this group is the pharmacological treatment with antipsychotics, since these drugs can modulate saliva production. Although, at present, the choice of antipsychotic treatment is based mainly on clinical efficacy, providing knowledge related to the oral safety profile of these drugs would allow individualized treatment for each patient, fulfilling the objectives of rational drug prescription and providing psychiatric professionals with an important tool in therapeutic decision making. In this sense, the link between antipsychotic medication and oral adverse effects deserves further study. In the light of the available evidence provided in this review, it would be of interest to study the link between the anticholinergic burden and oral health implications. Other unexplored fields include the study of the antipsychotic induced glycaemic alterations and its contribution to oral disease or the involvement of the antimicrobial activity or certain antipsychotic drugs on the oral microbiota and thus, oral health.
